# A case report of paravertebral low-grade malignant fibrous myxoid sarcoma

**DOI:** 10.1097/MD.0000000000018800

**Published:** 2020-01-17

**Authors:** Ming-Ming Zhao, Chen-Si Li, Jun-Wang, Yuan-Pei Cheng, Dong-Xu Zhao

**Affiliations:** Department of Orthopedic Surgery, China-Japan Union Hospital of Jilin, University Changchun, Jilin, PR China.

**Keywords:** case report, LGFMS+

## Abstract

**Rationale:**

Low-grade malignant fibrous myxoid sarcoma (LGFMS) is a malignant tumor that originates from soft tissues and has specific clinical and histopathological characteristics. Paravertebral LGFMS is rarely reported.

**Patient concerns:**

A 60-year-old woman had pain in the lower back and right anterior thigh for more than 3 years.

**Diagnosis:**

Paravertebral LGFMS.

**Interventions:**

Tumor resection, vertebral canal decompression and pedicle screw fixation.

**Outcomes:**

The tumor was excised, and the vertebral arch was fixed with pedicle screws at the root. Chemoradiotherapy was not performed. Her postoperative visual analogue scale (VAS) score decreased from 7 points at admission to 2 points at follow-up. The patient was discharged at postoperative day 13, and no recurrence was observed at the 6-month follow-up.

**Lessons:**

Although LGFMS is rare, it should be considered in differential diagnosis of other soft tissue tumors to avoid misdiagnosis and inappropriate treatment.

## 1 Introduction

Low-grade malignant fibrous myxoid sarcoma (LGFMS) was first reported in 2 patients by Evans and proposed as a new class of independent soft tissue tumors in 1987.^[1]^ LGFMS often occurs in the soft tissue of limbs, especially the lower extremities, whereas lesions in the head, chest, abdomen, intestine, and perineum are rarely reported.^[2]^ LGFMS occurs at all ages, and there are no clear statistics about its incidence. Immunohistochemistry plays an important role in the diagnosis of LGFMS. In most cases, gene fusions of t (7; 16) (q34; p11), FUS-CREB3L1 and FUS-CREB3L2 are present, and MUC4 staining is significant in diagnosis.^[5]^ The case of a patient with paravertebral LGFMS is reported herein. The relevant literature was reviewed, and clinical manifestations, pathological features, immunohistochemistry, differential diagnosis and treatments of LGFMS are discussed to improve the understanding of the disease.

## 2 Ethical review

Ethical review was nonessential because this case report did not violate the patient's privacy. Informed written consent was obtained from the patient for publication of this case report and accompanying images.

## 3 Case report

### 3.1 General information

The patient was a 60-year-old woman with pain in the lower back and right anterior thigh for more than 3 years. At the age of 12 years, painless nodules were discovered in the patient's lower back. At 32 years old, several egg-sized masses accompanied lower-back pain; the patient was diagnosed with fibromatosis, and the lesions were surgically removed in a local hospital. Restrained by medical capabilities at that time, pathological examination was not performed, and the surgical resection scope was limited; thus, the tumor was not completely removed. The patient complained that in the past 3 years, the pain level of the mass in her lower back increased and was accompanied by pain in the right anterolateral thigh. Physical examination revealed a palpable mass of approximately 18 × 8 cm in size at the L1-S1 level. Palpation further showed positive tenderness at the protruding site and decreased shallow sensation of the right anterolateral thigh. The muscle strength and reflex of both lower limbs were normal, and pathological signs were negative. There was a normal pulse in the arteria dorsalis pedis.

**Figure 1:**
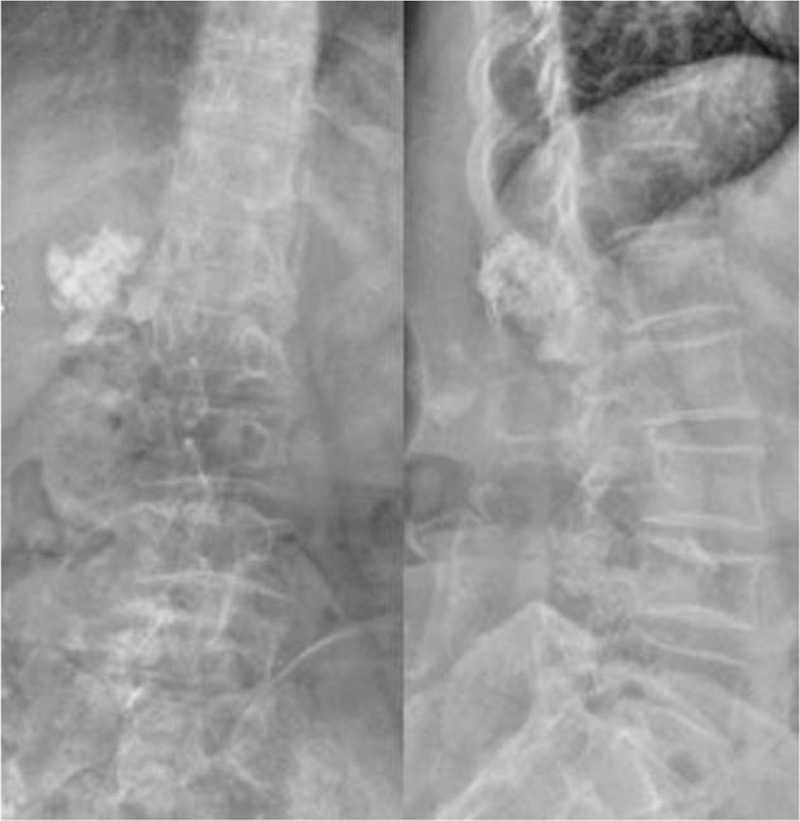
X-ray examination showed an irregular, high-density shadow in the posterior aspect of L1-2.

**Figure 2:**
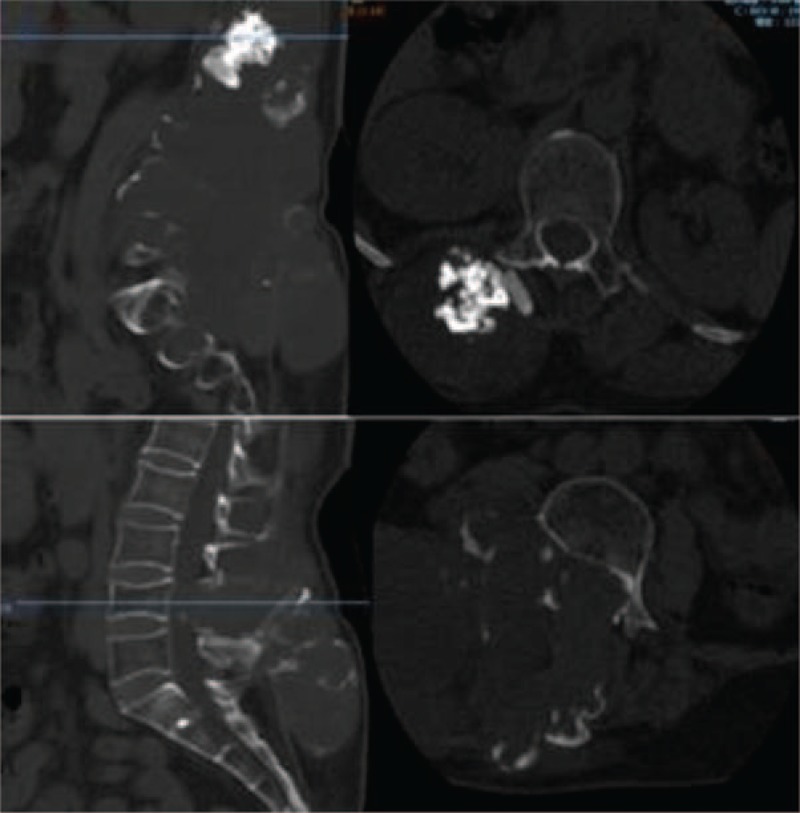
A CT scan revealed bone destruction in the adnexa of L1-S2 that was surrounded by lump-like abnormal density shadows. The CT intensity was approximately 9-53 HU. Multiple plaque-like calcifications ranging from T11 to S3 were also observed. CT = computed tomography.

**Figure 3:**
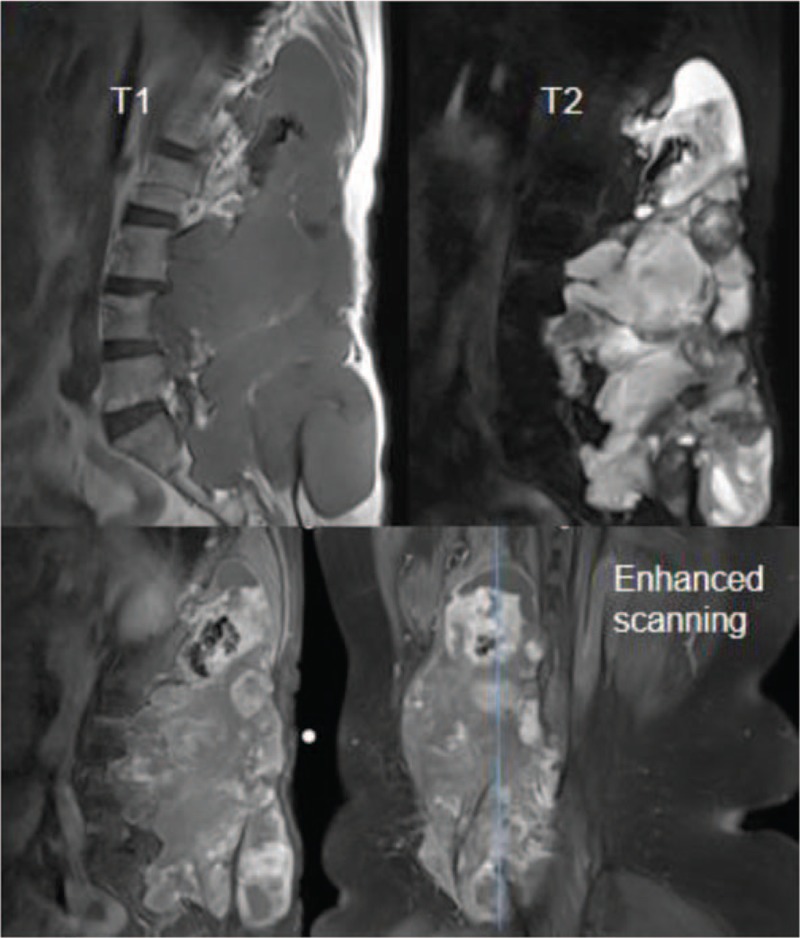
Magnetic resonance imaging showed a huge irregular mass shadow, with an unclear boundary of approximately 11 × 11 × 22 cm in size. There were uneven signals: low T1 signals were mixed with high T2 signals; multiple divisions were seen inside the mass; and multiple patchy high T1 and low T2 signals were present above the lesions. An enhanced scan indicated uneven reinforcement and obvious enhancement of the divisions in the lesions.

**Figure 4:**
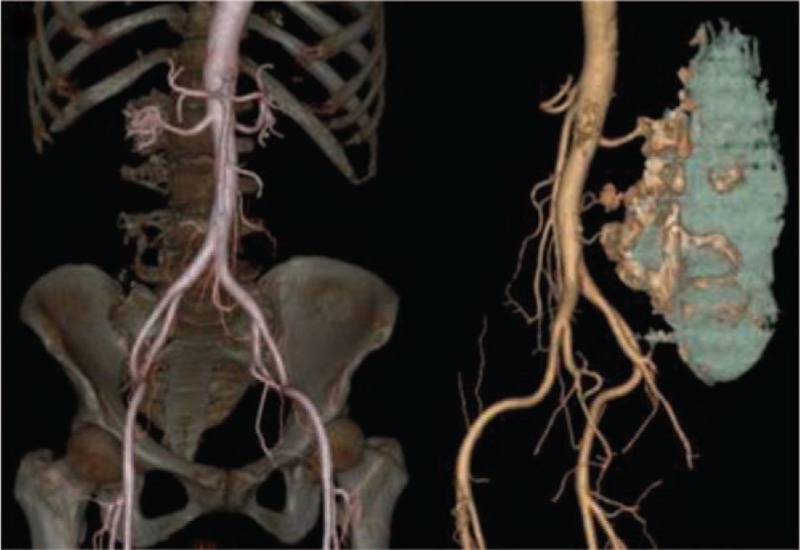
Abdominal aorta computed tomography angiography examination showed that 2 and 3 right lumbar arteries were on the lesion surface, with some small branches entering the lesion.

Auxiliary examination was also conducted. X-ray examination showed an irregular, high-density shadow in the posterior aspect of L1-2 (Fig. 1). A computed tomography (CT) scan revealed bone destruction in the adnexa of L1-S2 that was surrounded by lump-like abnormal density shadows. The CT intensity was approximately 9-53 HU. Multiple plaque-like calcifications ranging from T11 to S3 were also observed (Fig. 2). magnetic resonance imaging (MRI) showed a huge irregular mass shadow, with an unclear boundary of approximately 11 × 11 × 22 cm in size. There were uneven signals: low T1 signals were mixed with high T2 signals; multiple divisions were seen inside the mass; and multiple patchy high T1 and low T2 signals were present above the lesions. The lesions protruded into the abdominal cavity, the spinal canal and the posterior soft tissues, with protrusion into the abdominal cavity on the right side at the L3-5 vertebral level and into the intraspinal canal via the right intervertebral foramen, and the canal at the corresponding level was compressed. An enhanced scan indicated uneven reinforcement and obvious enhancement of the divisions in the lesions (Fig. 3). Abdominal aorta computed tomography angiography examination showed that 2 and 3 right lumbar arteries were on the lesion surface, with some small branches entering the lesion (Fig. 4). Ultrasound-guided puncture revealed spindle cell tumor pathology and partial myxoid degeneration and suggested a mesenchymal tissue origin. No chest metastasis was found by chest CT.

Her admission diagnosis was a paravertebral substantial mass (nature to be examined).

### 3.2 Treatments

Posterior lumbar tumor resection, decompression, iliac bone graft fusion and internal fixation were performed. A 30-cm incision was made at the median line of the mass. The subcutaneous tissue and deep fascia were cut layer by layer, and the tissues attached to the tumor capsule were slowly separated; the tumor supply blood vessels were then ligated, and the tumor pedicle was cut off, enabling removal of the tumor tissue. The tumor was solid and soft with myxoid tissue on the surface. Other tumor tissues were removed by the same surgical procedure. Muscle was dissected along the lamina and pulled laterally to expose L2, L3, L4, L5, S1 and bilateral facet joints. Seven appropriate pedicle screws were inserted at the left L2, L3, L4, L5, S1 and the right L2 and S1. Part of the vertebral plate between the L4 intervertebral plates was incised to relieve spinal cord compression. The bilateral facet joints of L2, L3, L4, and L5 were removed, and the cancellous bone was exposed as a bone graft bed. The allogeneic bone was trimmed into fragments and implanted into the bone graft bed. After washing, a drainage tube was placed, and the tissues were sutured layer by layer.

According to postoperative pathology, the mesenchymal tissue-derived low-grade malignant tumors were diagnosed as LGFMS. Immunohistochemistry results were as follows: vimentin (+), Dog-1 (+), CD34 (vascular +), CD99 (+), bcl-2 (+), Ki-67 (2% +), CD117 (−), s-100 (−), SMA (−), desmin (−).

**Figure 5:**
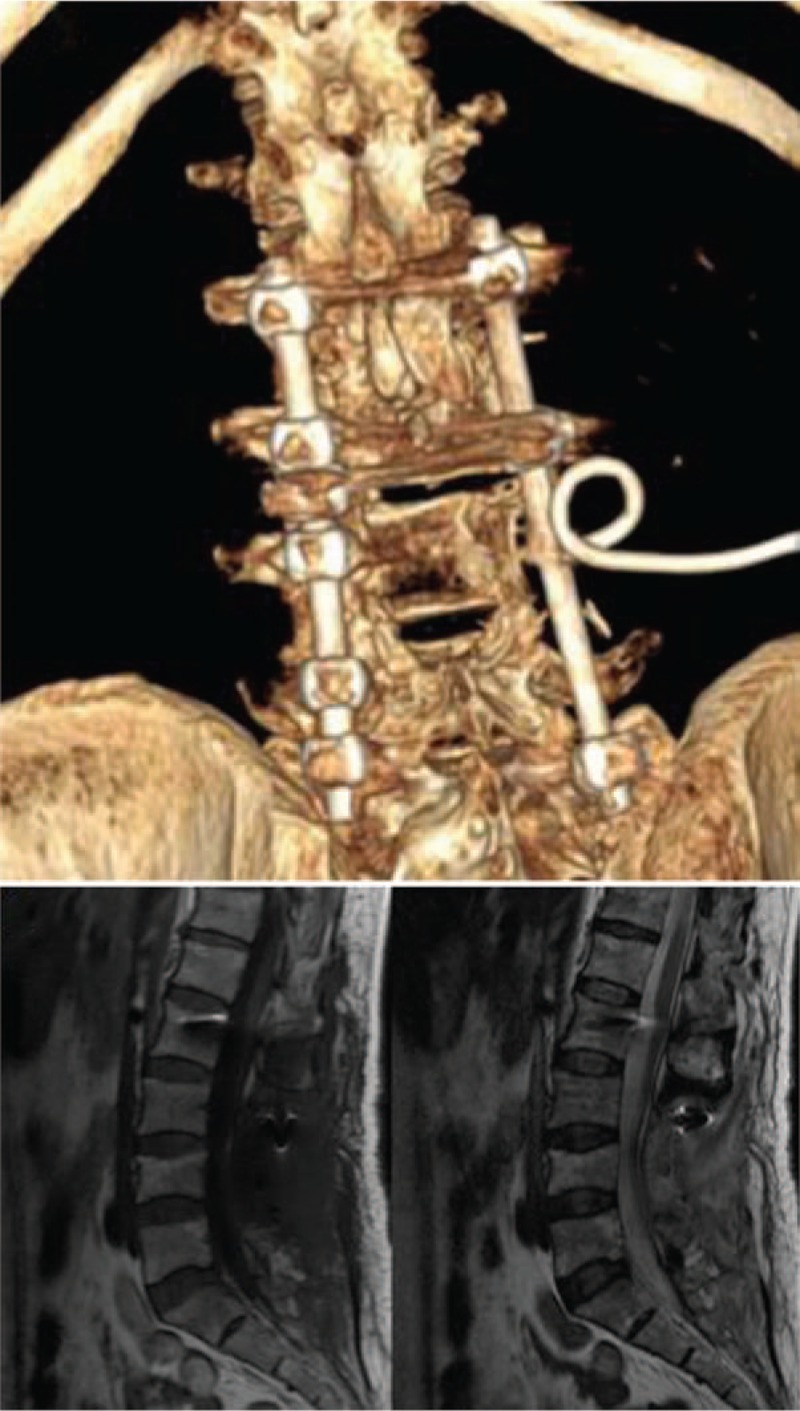
(A-B) The patient did not show recurrence at the 6-month follow-up.

Supportive treatment and anti-inflammatory treatment were provided after the operation; chemoradiotherapy was not performed. Her VAS decreased from 7 points at admission to 2 points at postoperative follow-up. The patient was discharged on postoperative day 13 and did not show recurrence at the 6-month follow-up (Fig. 5A and B).

## 4 Discussion

Low-grade malignant fibrous myxoid sarcoma (LGFMS) is a rare and malignant tumor that has a deceptively mild presentation. LGFMS often affects young people, though sometimes children and the elderly, and the lesions are commonly found in deep soft tissue. As the tumor of the patient described in this paper grew near the vertebrae and protruded into the abdominal cavity, it initially presented as painless mass. However, with the increase in tumor size, the dural sac was compressed forward and the soft tissue backward; accordingly, the patient gradually presented nerve symptoms involving right thigh pain and lower back pain.

LGFMS, with an alternative distribution in the fibrous area and myxoid area, is generally not observed on X-ray. On CT, the fibrous area is usually in equal density and the mucous area in low density. MRI has unique advantages for determining LGFMS, where the fibrous area shows low T1 and T2 signals, and the mucous area presents low T1 and high T2 signals. In the enhanced mode, the fiber area is slightly enhanced, though the mucous area is significantly enhanced.^[4]^ In our case, the tumor presented heterogeneous manifestations on T1 and T2 scans and high T1 and low T2 signals, which may be related to internal tumor hemorrhage. The histological features of LGFMS are mild, and common imaging methods, such as X-ray, CT, and MRI, have no specificity for LGFMS. Therefore, it is easy to confuse LGFMS with other benign or low-grade malignant soft tissue tumors, making LGFMS difficult to diagnose. Pathological examination is the gold standard for the diagnosis of this tumor.^[5]^ Microscopically, the cells in the tumor tissue are crowded and fused with spindle cells. As a type of mesenchymal tumor, LGFMS is relatively nonspecific with regard to immunohistochemistry. Most tumor cells are strongly positive for vimentin; CD99 and Bcl-2 are also expressed in some cells. However, CD34, CD56, S-100, CD117, desmin, β-catenin and cytokeratin are negative, and the proliferation index of Ki-67 is usually lower than 2%.^[6]^ Such findings are helpful for distinguishing myxofibrosarcomas from other soft tissue sarcomas. In our case, the Ki-67 proliferation index was over 2%, which was also consistent with the proliferation characteristics of most malignant tumors. Other immunohistochemical results basically matched those reported in the literature. MUC-4 is an immunostaining marker with relatively higher sensitivity and specificity for LGFMS. In addition, 2 specific chromosomal translocations are associated with LGFMS, t (7; 16) (q34; P11) or t (11; 16) (p11; P11), which lead to fusion of the FUS-CREB3L1 and FUS-CREB3L2 genes. This special immune marker is helpful for the diagnosis of LGFMS.^[3]^

Regarding the supply blood vessels of the tumor, Harish et al^[7]^ stated that the distribution pattern of blood vessels on the surface of the tumor can explain the enhancement mode of the lesion, that is, early peripheral enhancement and centripetal filling with time delay. However, Fujii et al^[8]^ reported an enhanced pattern of intestinal LGFMS in one case, where the myxoid area was significantly enhanced, indicating that the mode of enhancement was related to the dense cell distribution and fiber composition. The feeding vessels in our case originated from the second and third branches of the lumbar artery. Abdominal aortography showed that the tumor was gradually enhanced from the arterial stage to the venous stage, and the feeding vessels in the tumor could be seen at the arterial stage. Therefore, the enhancement pattern of the tumor in this case, namely, delayed centripetal filling and enhancement, may be related to the distribution of capillaries.

LGFMS tumors have a tendency to relapse and metastasize, usually to the lungs. A retrospective analysis of 54 LGFMS patients by Evans in 2000 found that local recurrence in 9% and distant metastasis in only 3%. In 2011, Evans surveyed 33 LGFMS patients with metastatic disease and found metastasis to the lungs, pleura and chest wall, and occasionally to the pericardium, in 15 cases. The interval of distant metastasis was as long as 45 years, with a median of 5 years.^[9,10]^ The patient in the current study had a history of nearly 50 years since the tumor was found at the age of 12, and no metastasis was found on chest CT examination. Although it grew in the paravertebral region, the tumor did not metastasize to the vertebral body but only compressed it, and this compression fully expressed the inertia of the tumor. Thus, although this tumor type has the potential to metastasize, a patient can survive decades after the initial surgery.

LGFMS can be distinguished from several diseases, such as fibromatosis, schwannoma, neurofibromas and myxofibrosarcoma, which do not contain “collagen rose”, a feature in LGFMS. This feature was first described in 1997, but its significance and pathogenesis in LGFMS remain unclear.^[11]^ Another characteristic of LGFMS is homology with hyalinizing spindle cell tumor with giant rosettes (HSCT), which is associated with same fusion gene and translocation. Some believe that HSCT is a variant of LGFMS with a larger collagen wreath, whereas others claim that the 2 types of tumors can be separated by the presence of epithelioid cells in HSCT. Comparatively, there are few reports of HSCT metastasis, indicating that HSCT has a relatively good prognosis compared with the that of LGFMS.^[12]^

Due to the inertia of LGFMS, surgery plays an important role in its treatment, and a negative surgical margin is an important factor affecting the prognosis. Because the malignancy and mitotic rate of LGFMS are low, LGFMS is expected to be less sensitive to chemoradiotherapy.^[6]^ Although there was no metastasis to the vertebral body in this case, its compression of the vertebral body and attachments caused bone destruction. After tumor excision, the vertebral body was fixed with pedicle screws, bilateral facet joints were removed, and cancellous bone was exposed as the bed for bone grafting to avoid overall instability of the spine.

## Acknowledgments

All the patients included in this article and all medical personnel involved in diagnosis and treatment of the disease are thanked.

## Author contributions

**Conceptualization:** Ming-Ming Zhao.

**Formal analysis:** Chen-Si Li.

**Investigation:** Yuan-Pei Cheng.

**Resources:** Jun Wang, Dong-Xu Zhao.

**Software:** Dong-Xu Zhao.

**Supervision:** Dong-Xu Zhao.

**Writing – original draft:** Ming-Ming Zhao.

**Writing – review & editing:** Ming-Ming Zhao.

Ming-Ming Zhao orcid: 0000-0002-8442-7153.
